# Biomarker Discovery by Sparse Canonical Correlation Analysis of Complex Clinical Phenotypes of Tuberculosis and Malaria

**DOI:** 10.1371/journal.pcbi.1003018

**Published:** 2013-04-18

**Authors:** Juho Rousu, Daniel D. Agranoff, Olugbemiro Sodeinde, John Shawe-Taylor, Delmiro Fernandez-Reyes

**Affiliations:** 1 Helsinki Institute for Information Technology, Department of Information and Computer Science, Aalto University, Espoo, Finland; 2 Childhood Malaria Research Group, College of Medicine, University of Ibadan, University College Hospital, Ibadan, Nigeria; 3 Department of Infectious Diseases and Immunity and Wellcome Trust Centre for Clinical Tropical Medicine, Imperial College London, London, United Kingdom; 4 Division of Parasitology, MRC National Institute for Medical Research, The Ridgeway, London, United Kingdom; 5 Department of Computer Science, University College London, London, United Kingdom; Institute for Systems Biology, United States of America

## Abstract

Biomarker discovery aims to find small subsets of relevant variables in ‘omics data that correlate with the clinical syndromes of interest. Despite the fact that clinical phenotypes are usually characterized by a complex set of clinical parameters, current computational approaches assume univariate targets, e.g. diagnostic classes, against which associations are sought for. We propose an approach based on asymmetrical sparse canonical correlation analysis (SCCA) that finds multivariate correlations between the ‘omics measurements and the complex clinical phenotypes. We correlated plasma proteomics data to multivariate overlapping complex clinical phenotypes from tuberculosis and malaria datasets. We discovered relevant ‘omic biomarkers that have a high correlation to profiles of clinical measurements and are remarkably sparse, containing 1.5–3% of all ‘omic variables. We show that using clinical view projections we obtain remarkable improvements in diagnostic class prediction, up to 11% in tuberculosis and up to 5% in malaria. Our approach finds proteomic-biomarkers that correlate with complex combinations of clinical-biomarkers. Using the clinical-biomarkers improves the accuracy of diagnostic class prediction while not requiring the measurement plasma proteomic profiles of each subject. Our approach makes it feasible to use omics' data to build accurate diagnostic algorithms that can be deployed to community health centres lacking the expensive ‘omics measurement capabilities.

## Author Summary

Many infectious diseases such as tuberculosis and malaria are challenging both for scientists trying to understand the biochemical basis of the diseases and for medical doctors making diagnosis. The challenges arise both from the dependence of the diseases on sets of proteins and from the complexity of the symptoms. Biomarkers denote small sets of measurements that correlate with the phenotype of interest. They have potential use both in advancing the basic biomedical research of infectious diseases and in facilitating predictive diagnostic tools. We propose a new method for biomarker discovery that works by finding canonical correlations between two sets of data, the plasma proteomic profiles and clinical profiles of the subjects. We show that the method is able to find candidate proteomic biomarkers that correlate with combinations of clinical variables, called the clinical biomarkers. Using the clinical biomarkers improves the accuracy of diagnostic class prediction while not requiring the expensive plasma proteomic profiles to be measured for each subject.

## Introduction

The aim of biomarker discovery is to find small subsets of measurements in ‘omics data that correlate with the clinical syndromes or phenotypes of interest. Despite the fact that most clinical phenotypes (e.g. diseases) are characterized by a complex set of clinical parameters, with a variable degree of overlap, current computational approaches do not take into consideration the multivariate nature of the phenotypes. The challenges arise both from the dependence of the diseases on several proteins and from the complexity of the symptoms. To overcome this limitation, in our framework, the data to be analysed is represented by two views, namely a plasma proteomics profile, and a set of clinical data composed of patient history, signs, symptoms and clinical laboratory measurements of the individuals with syndromes of interest. This type of problem can be described as multivariate in both the views, and the aim is to discover a sparse set of ‘omic variables (proteomic-biomarkers) that correlates with a combination of clinical variables (clinical-biomarkers).

Given the typically high number of variables and small number of patient samples in clinical ‘omic studies, dimensionality reduction techniques such as Principal component analysis (PCA) and Canonical Correlation Analysis (CCA) have become popular. PCA allows one to discover a set of latent variables in the data that explain most of the variance but they may not correlate with the clinical syndrome of interest. In contrast, CCA performs dimensionality reduction for two co-dependent datasets simultaneously so that the latent variables extracted from the two datasets are maximally correlated. Thus, the latent variables computed from one of the datasets can be used to predict the ones computed from the other, which is the basic goal in biomarker discovery. However, in both PCA and CCA, the latent variables depend on all variables and therefore hinder clinical interpretation and biomarker discovery and validation. To address these computational limitations sparse variants of PCA (SPCA) and CCA (SCCA) have been independently developed [1]–[3]. These methods use a *L1*-norm penalization to variable weights which favours models with a small set of variables having a non-negligible weight. Sparse approaches impose the penalisation in both views of the data, thus generating latent variables depending on small sets of variables [2], [3]. Recently we have proposed an asymmetrical algorithm that imposes sparsity only in one of the data views [1] by penalizing dual variables related to the latent variables of the other dataset. This asymmetrical approach favours latent variables that relate to small clusters of data points.

Here we use our asymmetrical SCCA algorithm for the unsupervised discovery of candidate biomarkers by correlating plasma proteomics data to multivariate clinical parameters from two human infectious diseases namely, tuberculosis and malaria, which present with overlapping complex clinical phenotypes in the affected host.

Tuberculosis is the leading bacterial cause of death worldwide, with an estimated 8.8 million new cases of active disease and 1.6 million deaths per year [4]. The global burden of TB occurs in a background of complex disease phenotypes that range from the presence of latent TB to respiratory and constitutional symptoms overlapping with those of pulmonary active TB. Latent TB infection is thought to affect one third of the world's population and a higher proportion of the population of TB-endemic areas [5]. In this scenario the challenge is to distinguish symptomatic patients with active TB from those with latent disease but whose presenting symptomatology is attributable to some other infectious or inflammatory process [6].

Cerebral malaria (CM) and severe malarial anemia (SMA) are the major severe disease syndromes in African children with a high level of mortality in the under-five age group. The current WHO case definitions for severe malaria combine *P. falciparum* blood stage parasitemia with coma, severe anemia or respiratory distress [7], and it is well documented that there is significant overlap across these syndromes [8].

To validate the biomarkers discovered by the SCCA approach, we study the prediction of diagnostic classes using the biomarkers. In particular we study a scenario were the expensive proteomics data is only available during the training time of the models, whilst in prediction time, clinical data and a previously learned biomarker model is available. In our belief this is a realistic setup considering possible real-world deployment of decision support systems into resource-poor health care centres.

## Materials and Methods

### Datasets

The active TB dataset [6] consists of 412 patient data with three datasets: serum proteome profiles measured by SELDI-ToF mass-spectrometry [6], [9] (270 variables), clinical data (19 variables) and diagnostic classes (Active TB, Symptomatic Control, Asymptomatic Control).

The childhood severe malaria dataset consist of 944 patient data with three datasets: plasma proteome profiles measured by mass-spectrometry (774 variables), clinical data (57 variables) and diagnostic classes (Cerebral Malaria (CM), Severe Malaria Anaemia (SMA), Uncomplicated Malaria (UM), Disease Control (DC) and Community Control (CC)).

**Figure 1:**
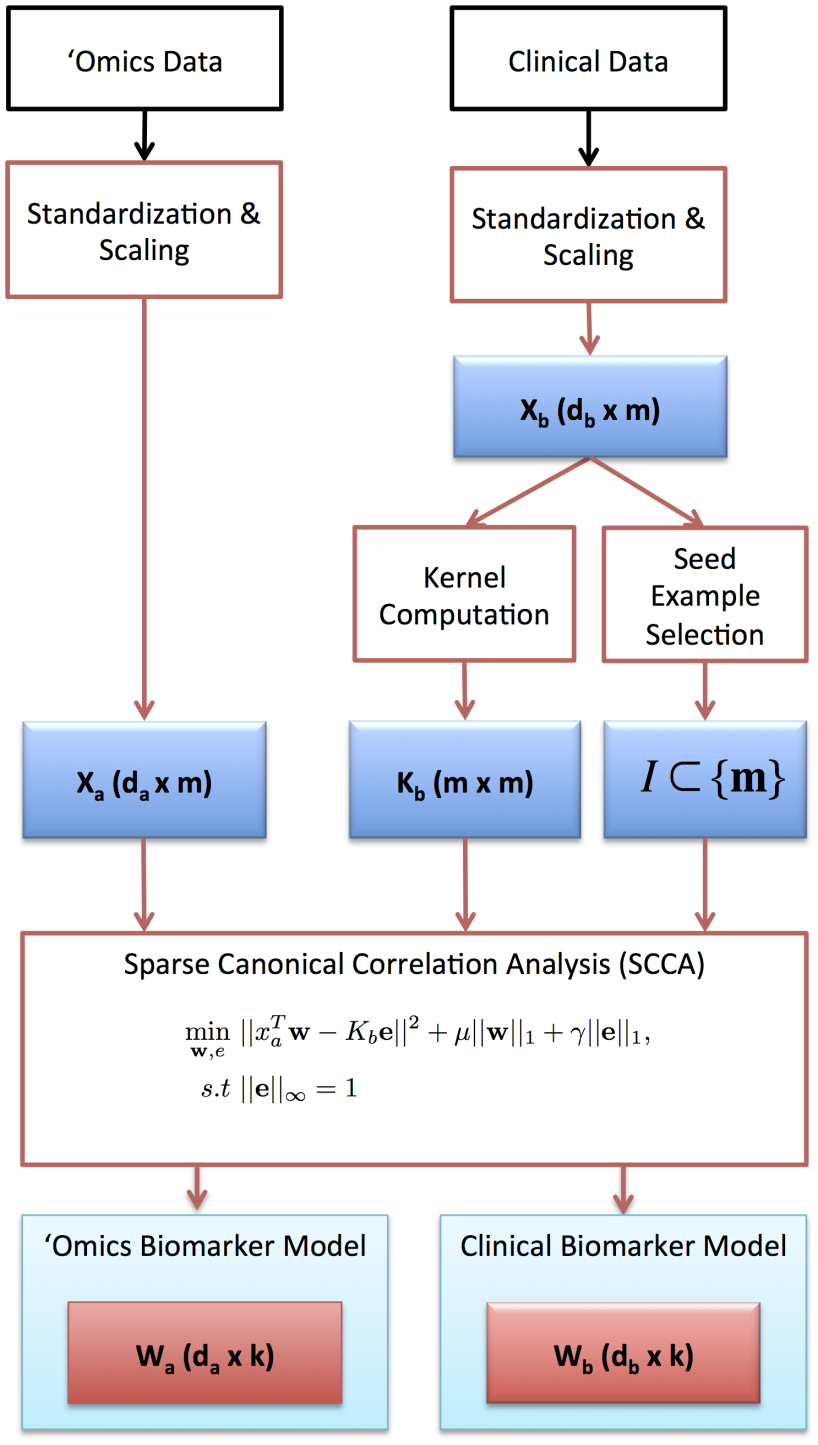
Algorithm framework for biomarker extraction.

Plasma was subjected to high-throughput proteomic profiling by mass spectrometry as previously described [6], [9]. The proteomics and clinical variables were standardized by subtracting the mean and dividing by standard deviation. Standardized proteomics and clinical profiles were converted to unit norm vectors by dividing by the Euclidean norm (Figure 1).

### Biomarker extraction by sparse canonical correlation analysis

Figure 1 shows a workflow of the algorithmic and experimental framework used for biomarker extraction. We assume data in two views, represented by *d_a_ x m* matrix *X_a_ = (x_a_^(1)^,…,x_a_^(m)^)* and *d_b_ x m* matrix *X_b_ = (x_b_^(1)^,…,x_b_^(m)^)*, where *d*_a_ and *d_b_* are the dimensions of the feature vectors in the two views. The *i*'th example is thus given by a pair *(x_a_^(i)^,x_b_^(i)^)*.

Canonical correlation analysis (CCA) is a family of statistical algorithms designed to situations where there are two available views or measurements of the same phenomenon and the goal is to find latent variables that explain both views (‘the generating model’) [10]. The CCA algorithm aims to find projection directions *w_a_* and *w_b_* that maximize the correlation of the projected data, the scores *s_a_^(i)^ = w_a_^T^x_a_^(i)^* and *s_b_^(i)^* = *w_b_^T^x_b_^(i)^*, in the two views:
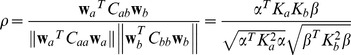
where *C_ab_ = X_a_X_b_^T^* is the empirical covariance matrix of the views over the dataset. The first expression gives the correlation computed with explicit feature weights. The second expression gives the dual representation in terms kernel matrices *K_a_ = X_a_^T^ X_a_* and K_b_ = *X_b_^T^ X_b_, as well as dual variables α = (α_1_,…, α_m_) and β = (β_1_,…,β_m_) giving weights to the examples in the two views*. The corresponding projection directions are given in the dual representation by linear combinations of examples, *w_a_ = X_a_α and w_b_ = X_b_β*. When CCA is performed in dual representation, it is commonly called Kernel Canonical Correlation Analysis (KCCA).

CCA can be applied to the data iteratively via deflation, that is, the projection of the data to the orthogonal complement of *w_a_* and *w_b_*, respectively, and performing the CCA analysis for the projected data. This process results in a sequence of projections (*w_a_(1)*, *w_b_(1))*, *(w_a_(2)*, *w_b_(2)),…*, *(w_a_(r)*, *w_b_(r))*, where *r* denotes the minimum of the numerical ranks of *X_a_ and X_b_*. The resulting projections are orthogonal to each other: *w_c_(i)^T^w_c_(j) = 0* if *i≠j*, where by *c* we denote either of the two views *a* or *b*. We call the pair (*w_a_(1)*, *w_b_(1))*, the leading pair of projections. By definition, the leading pair has the maximum canonical correlation in the sequence. The sequence of CCA projections is compactly represented by the matrices *W_a_ = (w_a_^(1)^,…, w_a_^(r)^)* and *W_b_ = (w_b_^(1)^,…, w_b_^(r)^)*.

Sparse canonical correlation analysis differs from KCCA in that it aims to find sparse projection directions, those that only depend on a small number of variables. We use an asymmetric formulation of SCCA [1], where sparse projection directions are aimed for in one view, while in the other view dual sparsity is aimed for, meaning that each projection direction can be expressed as a linear combination of a small number of examples. The optimization to be solved is the following:
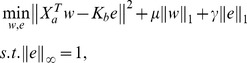
where *w* denotes the projection direction in the first view, and *e* denotes a vector of dual coefficients, where the coefficient *e_k_, k = 1…m*, denotes the weight of *x_b_^(i)^*.

The first term of the objective aims to make the projection scores s_a_ = w_a_ *^T^X_a_* and *s_b_ = K_b_e* to match, the second term penalizes the feature weights in the first view by 1-norm (thus imposing sparsity), and the final term penalizes the dual variables by 1-norm (thus imposing dual sparsity). The infinity norm in the constraint ensures that at least one example will have non-zero dual coefficient. This is achieved by fixing one example, indexed by *k*, to have coefficient *e_k_ = 1* and allowing the rest of the coefficients *e_l_*, *l≠k* vary. The fixed example is called the seed example. The SCCA hyperparameters *μ* and *γ* control the balance between primal and dual sparsity, in the respective views. We used *μ = 1* and *γ = 1* in our experimental framework.

SCCA can be applied iteratively to extract a series of projection directions by using one of two alternative approaches. In the deflation approach, like in CCA, deflation is used to arrive at a sequence *(w^(1)^,e^(1)^), (w^(2)^,e^(2)^),…,(w^(r)^,e^(r)^)* of *r* pairs of projection directions that are orthogonal to each other. In other approach, by selecting a set of different seed examples {*s_1_,…,s_k_*} one obtains a set of *(w^(1)^,e^(1)^), (w^(2)^,e^(2)^),…,(w^(k)^,e^(k)^)* projection directions, which, in contrast to the deflation approach, are in general not orthogonal. The rationale of not requiring orthogonality of the components is two-fold: First, the deflation approach to generate orthogonal components is an order of magnitude slower method, due to the need to search for the best seed after each deflation operation. Secondly, when using the projection directions as input features to classification, there is no obvious benefit from the orthogonality. The non-deflating approach produces a set of latent variables that may have some redundancy due to near collinearity, however, machine learning methods such as SVM are not expected to be hampered by this. Indeed, in our tests, the SVM accuracy on SCCA features with and without deflation was very similar (data not shown).

Without loss of generality, we assume that the sequence is sorted so that the pair *(w^(1)^,e^(1)^)* has maximal canonical correlation in the sequence. Analogously to CCA, this pair is called the leading pair. In our experimental framework we selected seed examples by *k*-means clustering of the clinical data and choosing the cluster centers as the seeds. The value *k = 3* was used for number of clusters in the biomarker extraction experiment and the value *k = 30* in the diagnostic class prediction experiment.

### Diagnostic class prediction

**Figure 2:**
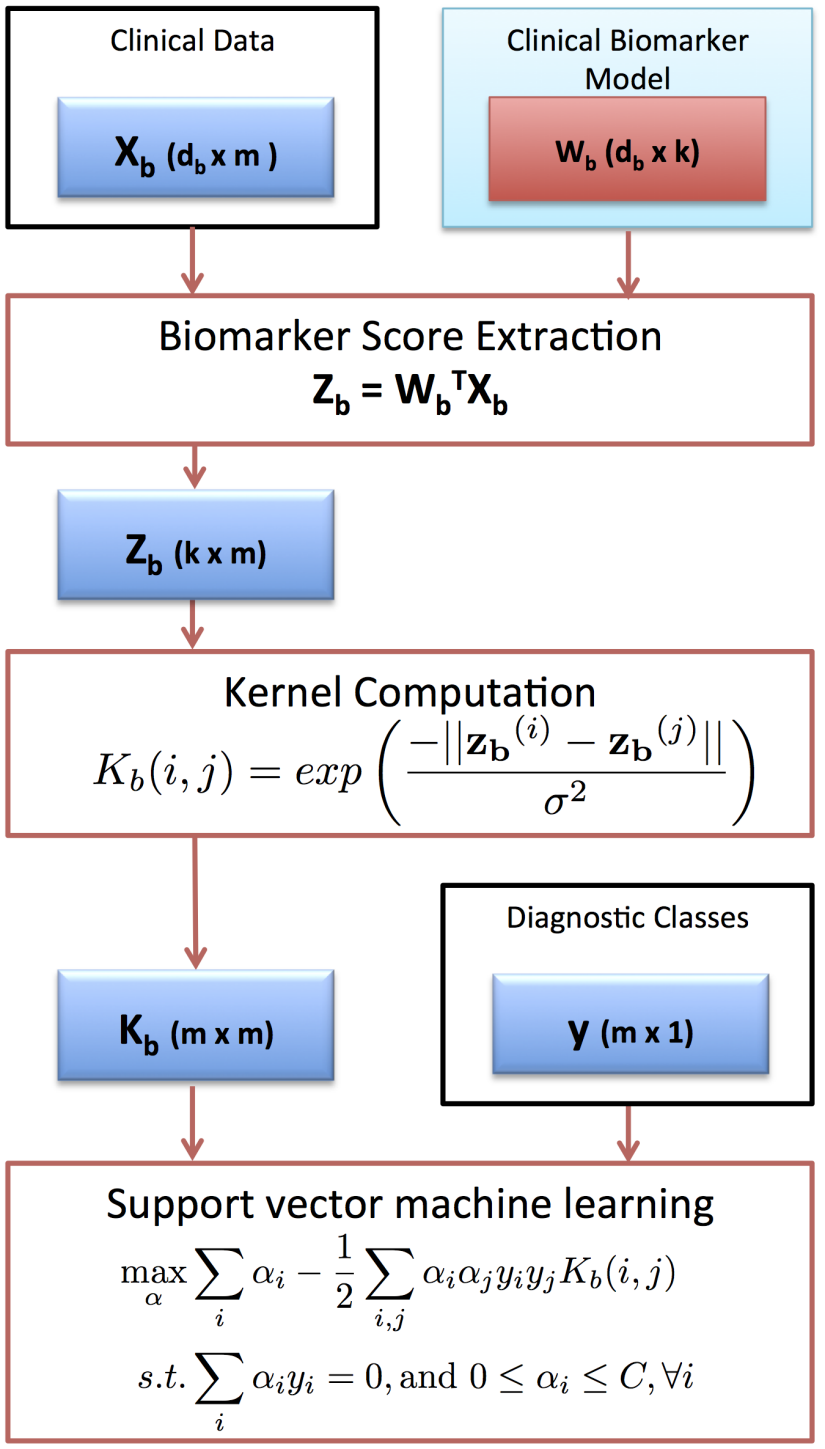
Algorithm framework for diagnostic class prediction.

Figure 2 shows the workflow in diagnostic class prediction. We assume as input clinical data and the biomarker model built by canonical correlation analysis. In the experiments we use a model with 60 projection directions, 30 from SCCA and 30 projection directions from KCCA. The clinical data is projected onto the biomarker model *W_b_* to obtain biomarker scores *z_b_^(i)^ = W_b_^T^*x_b_^(i)^ for each example. The Radial Basis Function (RBF) kernel
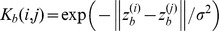
is used as a non-linear transformation of the biomarker scores. We trained a Support Vector Machine (SVM) to predict diagnostic classes by using the RBF kernel as input and the predefined diagnostic classes as the output. As the SVM requires a binary classification problem, we use a one-against-one scheme to train a separate classifier for each pair of diagnostic classes. For evaluation, 10-fold cross validation was used. The RBF kernel parameters and the margin softness of SVM were tuned by internal 10-fold cross-validation in each training fold.

We compared the CCA-based methodology to using raw clinical data as the input to SVM without making use of the biomarker model and using proteomics data processed with principal component analysis. We used 30 first principal components as the input to SVM.

### Statistical significance testing

Statistical significance of the results was estimated using randomization tests. In randomization, a background data distribution consistent with the null hypothesis is generated by simulation, where the statistical connection to be tested has been broken, but the data distribution is otherwise kept close to the original data. In the case of canonical correlation analysis, we want to test if the correlation of the two views is significant, we use the null hypothesis H0: The data *X_a_* and *X_b_* are not correlated”. To generate a sample of canonical correlation values consistent with the null hypothesis, we generated randomized versions of the data by permuting the rows of the data matrix *X_b_*, and performed canonical correlation analysis for the randomized data. This process was repeated 500 times to generate a set of canonical correlation values representing the distribution under the null hypothesis. The significance level of the test is given by the fraction of the distribution that is above the canonical correlation value obtained on the original data. We note that the randomization setup used here also automatically corrects for a possible multiple testing bias.

## Results

Firstly, we assessed the capabilities of SCCA for extracting biomarkers from data that is organized in two views, ‘omics data and clinical profile data. For this purpose, we used the leading pair of SCCA projection directions as it encapsulates the highest correlation between the ‘omics and clinical views. Secondly, we assessed the utility of the extracted biomarkers in predicting diagnostic classes. Specifically, we studied a scenario where we assume that ‘omics data is not available in prediction phase, but only in training phase.

### Tuberculosis dataset biomarker extraction using SCCA

**Figure 3:**
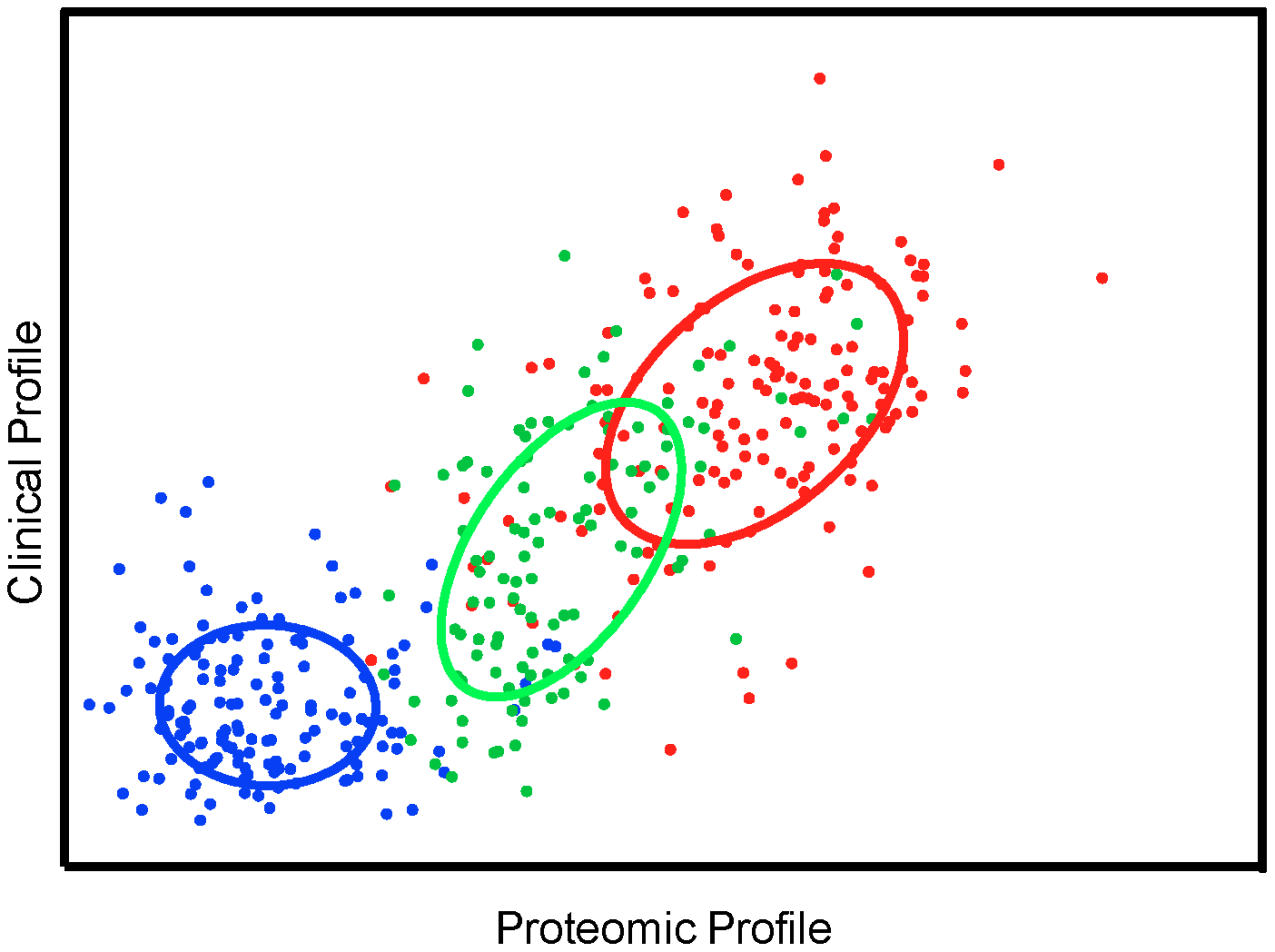
Sparse canonical correlation between proteomics and clinical profiles in the TB dataset. The x-axis gives the score in the proteomics profile while the y-axis gives the score in the clinical profile. Data points labeled based on the three diagnostic classes Active TB (red), Symptomatic Control (green) and Asymptomatic Control (blue). Ellipses denote the mean and covariance of the class clusters.

The leading pair of projection directions extracted from the proteomics (view *a*) and clinical data (view *b*) by sparse canonical correlation analysis showed a statistically significant correlation coefficient of 0.79 (0.01% significance level). Figure 3 depicts the correlation of the data in the directions corresponding to the leading pair of SCCA projections. The class clusters are relatively tight and do not overlap significantly. The leading pair of projection directions can be seen to pick up the properties in the data that separate active TB from symptomatic and asymptomatic controls.

**Figure 4:**
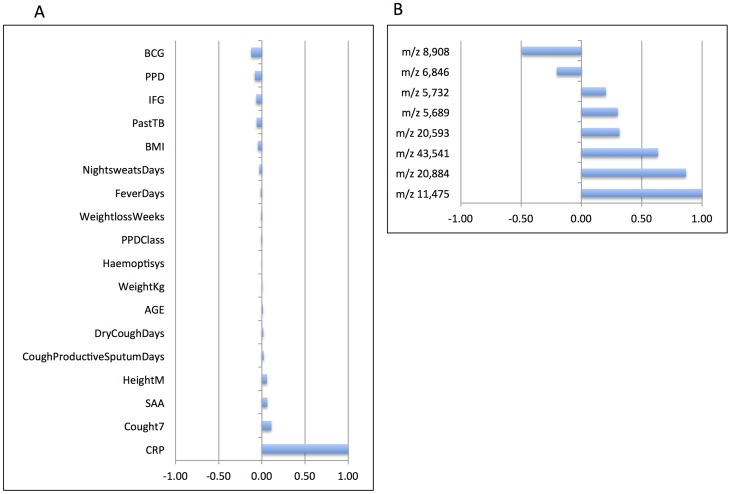
TB clinical variables (a.) and plasma proteome m/z clusters (b.) with non-negligible coefficients in the SCCA model.

The proteomic profile, given by the weights in the leading projection direction *w_a_^(1)^* (Figure 4) included 8 proteomic variables with non-negligible coefficients, selected out of 271 (3%), whilst in the clinical profile, given by the weights in *w_b_^(1)^*, 13 out of 18 variables had non-negligible coefficients. *C-reactive protein (CRP)*, has by far the largest positive weight in the clinical profile, persistent cough (*Cought7*), serum amyloid protein (SAA) are other discernible variables with positive weights, indicating that high values for them associate more frequently with active TB cases than the other two groups. *BCG vaccination*, *PPD skin test*, interferon-gamma level (*IFG*), and previously case of TB (*PastTB*) have discernible negative weights, and thus intuitively correspond to variables less often found among active TB cases than the other two groups. Body mass index (*BMI*) *and* height appear with opposite signs, consistent with the definition of BMI.

In the plasma proteomic profile the proteins at mass peaks *m/z* 11,475, *m/z* 20,884 and *m/z* 43,541 have the largest positive weights (presence makes the proteomic score higher). The protein at mass peak m/z 8,908 has the largest negative weight (absence makes the proteomic score higher).

### Malaria dataset biomarker extraction using SCCA

**Figure 5:**
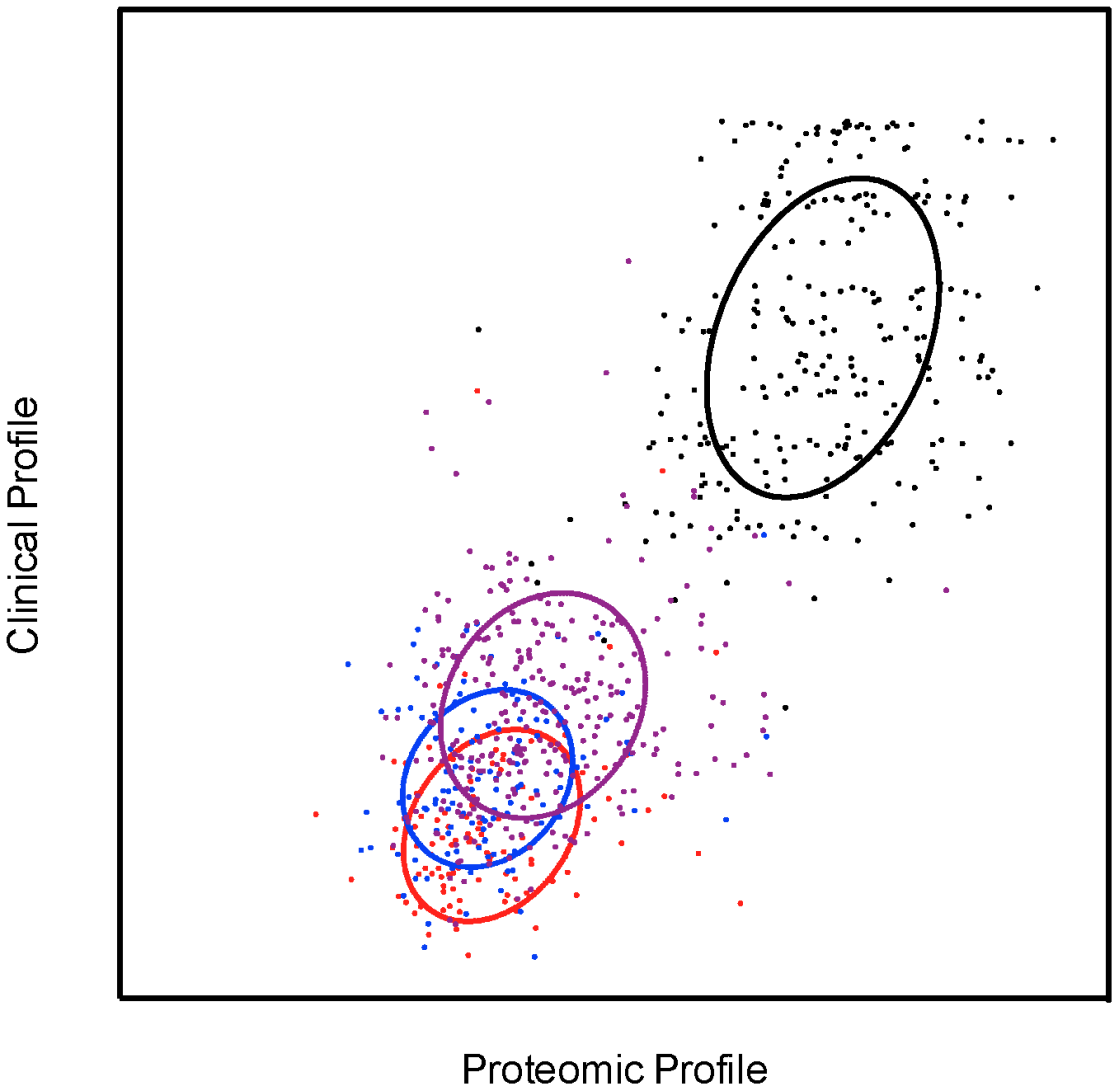
Sparse canonical correlation between proteomics and clinical profiles in the childhood severe malaria dataset. The x-axis gives the score in the proteomics profile while the y-axis gives the score in the clinical profile. Data points labeled based on the four diagnostic classes Community Controls (black), Uncomplicated Malaria (purple), Severe Malaria Anaemia (blue) and Cerebral Malaria (red). Ellipses denote the mean and covariance of the class clusters.

**Figure 6:**
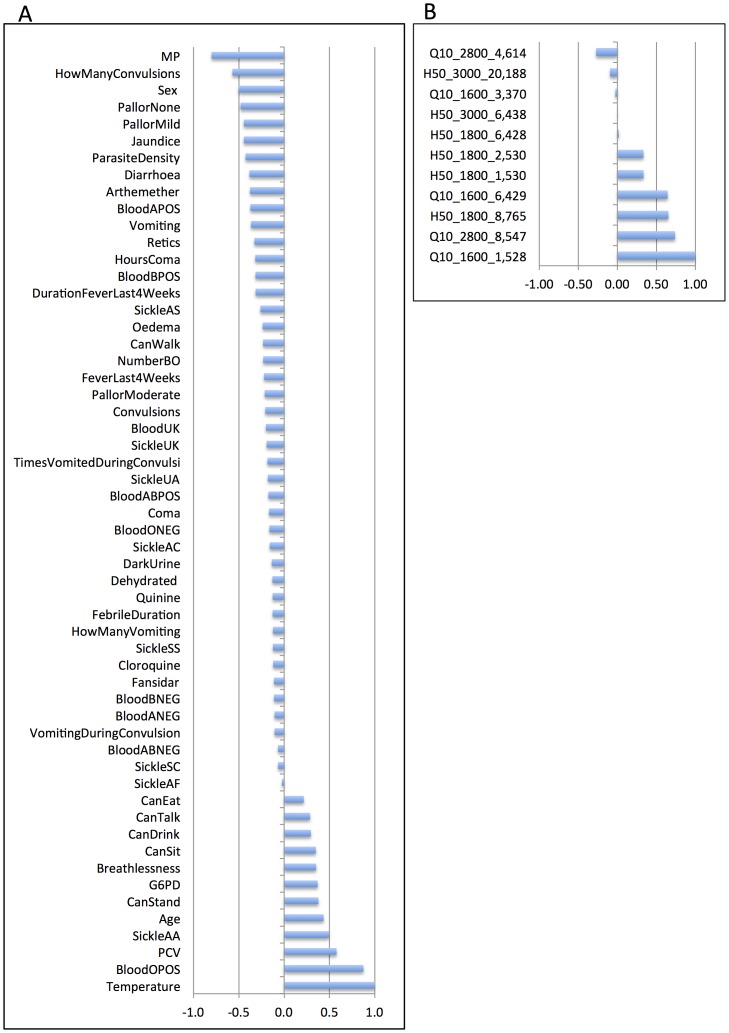
Malaria clinical variables (a.) and plasma proteome m/z clusters (b.) with non-negligible coefficients in the SCCA model.

Figure 5 depicts the projection of the malaria data onto the leading pair of SCCA directions. The canonical correlation showed a statistically significant correlation coefficient of 0.75, (0.01% significance level). The data projection clearly clustered non-malaria community controls (CC) apart from malaria cases *UM*, *SMA* and *CM*. Figure 6 shows the weights of the proteomic and clinical variables in the leading SCCA projection directions. Eleven proteomic variables out of 774 (1.4%) were present in the proteomic profile w_a_^(1)^, whilst all variables were present in the clinical profile w_b_^(1)^. The highest weights in the clinical profile, were *temperature*, and O-positive blood type (*BloodOPOS*). In addition, 10 other clinical variables had positive weights, roughly indicating more abundant among the community controls than the other classes. The highest negative weights are with presence of malaria parasites (*MP*) and the presence of convulsions (*HowManyConvulsions*). In addition, 42 other clinical variables had negative weights, thus intuitively less abundant among the community controls. In the proteomic profile, the proteins at mass peaks *m/z Q10_1600_1,528*, *Q10_2800_8,547* and *H50_1800_8,765* had the highest positive weights and in addition three other variables had positive weights (presence makes proteomic score higher). The proteins at mass peaks *m/z Q10_2800_4,614* and *H50_3000_20,188* had the highest negative weights (absence makes proteomic score higher).

### Diagnostic class prediction

Table 1 depicts the results of classification of the tuberculosis data in five classes defined by the latency and the presence of symptoms. We compared three different data: using proteomics data processed with principal component analysis (*PCA-Proteomics*), using clinical data only (*Raw-Clinical*) and using the clinical projections extracted by canonical correlation analysis (*K+SCCA-Clinical*). First, we observed that *K+SCCA-Clinical* had higher accuracy than *Raw-Clinical* in distinguishing non-TB subjects from symptomatic cases with no latent TB (85% vs. 77%) and from symptomatic cases with latent TB (90% vs. 79%) and in distinguishing non-TB subjects from non-symptomatic latent TB cases (85% vs. 78% accuracy). In the other classification tasks, the *K+SCCA* approach generally achieved similar level of accuracy as using the clinical data alone. In six of the experiments *PCA-proteomics* was the most accurate method, achieving 98% accuracy or higher. In contrast, in the other four experiments the accuracy of *PCA-Proteomics* ranges between 52%–77% and it is the least accurate method.

**Table 1:** Diagnostic class prediction in the TB dataset.

	PCA-Proteomics	Raw-Clinical	K+SCCA-Clinical
	ACC ± s.d.	ACC ± s.d.	ACC ± s.d.
Active TB vs. Symptomatic Latent TB	0.77±0.07	0.87±0.07	0.86±0.05
Active TB vs. Symptomatic No-Latent TB	0.76±0.05	0.90±0.04	0.91±0.04
Active TB vs. No-Symptomatic Latent TB	0.98±0.03	0.92±0.08	0.92±0.08
Active TB vs. No-Symptomatic No-Latent TB	0.99±0.02	0.94±0.04	0.94±0.04
Symptomatic Latent-TB vs. Symptomatic No-Latent TB	0.52±0.18	0.68±0.16	0.70±0.15
Symptomatic Latent-TB vs. No-Symptomatic Latent TB	1.00±0	0.74±0.14	0.75±0.15
Symptomatic Latent-TB vs. No-Symptomatic No-Latent TB	1.00±0	0.79±0.11	0.90±0.11
Symptomatic No-Latent TB vs. No-Symptomatic Latent TB	1.00±0	0.90±0.09	0.87±0.06
Symptomatic No-Latent TB vs. No-Symptomatic No-Latent TB	0.99±0.02	0.77±0.13	0.85±0.05
No-Symptomatic Latent TB vs. No-Symptomatic No-Latent TB	0.59±0.10	0.78±0.11	0.85±0.10

Table 2 shows similar experiments for the malaria data. Here, we considered one-against-one classification within five classes (*SMA*, *CM*, *UM*, *DC*, and *CC*). We first notice that *Raw-Clinical* obtained significantly higher accuracy than *PCA-Proteomics* alone for all class pairs. The *K+SCCA-Clinical* approach achieves higher accuracy than *Raw-Clinical* in two important cases: distinguishing *Severe Malaria Anaemia* from *Uncomplicated Malaria* (90% vs. 85%) and *Severe Malaria Anaemia* from *Disease Control* (97% vs. 92%). For the class pairs *CC* vs. *SMA*, *CC* vs. *UM* and *DC* vs *UM*, *Raw-Clinical* and the *K+SCCA-Clinical* achieve 99%–100% accuracy. For the pairs *CC* vs. *CM*, *CC* vs. *DC*, and *CM* vs. *UM*, the *K+SCCA-Clinical* approach is slightly inferior, and for the pairs *CM* vs. *DC* and *CM* vs. *SMA* significantly inferior to *Raw-Clinical*. We also noticed that the *K+SCCA-Clinical* approach was able to improve on the proteomics data in all cases.

**Table 2:** Diagnostic class prediction in the Malaria dataset.

	PCA-Proteomics	Raw-Clinical	K+SCCA-Clinical
	ACC ± s.d.	ACC ± s.d.	ACC ± s.d.
CC vs. CM	0.94±0.06	0.98±0.02	0.96±0.03
CC vs. DC	0.91±0.04	0.97±0.03	0.94±0.04
CC vs. SMA	0.98±0.04	0.99±0.02	0.99±0.02
CC vs. UM	0.92±0.03	0.99±0.01	0.99±0.02
CM vs DC	0.75±0.08	0.99±0.02	0.88±0.06
CM vs. SMA	0.61±0.12	0.88±0.07	0.76±0.08
CM vs UM	0.73±0.06	0.93±0.04	0.89±0.04
DC vs SMA	0.79±0.09	0.92±0.05	0.97±0.03
DC vs UM	0.79±0.06	1.00±0	0.99±0.02
SMA vs UM	0.71±0.07	0.85±0.05	0.90±0.05

## Discussion

In this paper, we have put forward an approach for discovering biomarkers from plasma proteomic data by canonical correlation to clinical data collected from the same subjects. We have also shown an approach to predict diagnostic classes based on the selected biomarkers.

We analysed a set of data consisting of plasma proteome and clinical profiles. Sparse canonical correlation analysis was shown to be effective in extracting small sets of proteomic variables, each representing a plasma protein, that correlate with clusters of similar clinical phenotypes in statistically significant manner (p-value 0.01). Sparsity of the extracted biomarker models is shown by the fact that 1.5% and 3% of the proteomics variables had non-negligible coefficients in the malaria and TB models, respectively. The sparsity of the ‘omic view of the SCCA model is deemed to be crucial for interpretation by human experts, as the set of proteomic variables to be studied remains tractable. Unlike SCCA, the KCCA method does not impose sparsity, so the KCCA is less amenable to human analysis.

In diagnostic class prediction, we show that via canonical correlation analysis, it is possible to make use of proteomic data in order to improve on the diagnostic classification, even if no proteomics data is available at the time of prediction, only at the time of training the model. This is a close match to a real-world scenario of deploying a diagnostic tool to health care centres without expensive ‘omics measurement capabilities.

In our experiments, the proposed approach appears to be advantageous (a) when proteomics data contains a strong signal predictive of the classification (i.e. PCA-Proteomics accuracy higher than Raw-Clinical) that can be mediated by the K+SCCA model, or (b) when proteomics data alone does not predict well (i.e. PCA-Proteomics accuracy lower than Raw-Clinical) but there is a synergistic latent signal between the proteomic and clinical profiles that K+SCCA can pick up. In the case of TB, a strong proteomics signal (case a) is found in six of the ten comparisons. In four of those six cases, K+SCCA matches the performance of Raw-Clinical, improves on Raw-Clinical on two of the cases and marginally loses in one of the cases. Evidence of a synergistic signal (case b) is found in four of the ten comparisons, in three of which K+SCCA matches Raw-Clinical and in one it exceeds the accuracy of Raw-Clinical: determining the presence of latent TB when there are no symptoms (85% accuracy versus 77% with clinical data alone). In malaria, first we note that the clinical data is very strong, there are no comparisons where PCA-proteomics exceeds the accuracy of Raw-Clinical (i.e. case a). Thus, in this data set, the K+SCCA is required to pick out a synergistic latent signal (case b) between the proteomic and clinical variables, in order to improve on the predictions from clinical data alone. This appears to take place in two experiments: in the separation of severe malaria anemia from both uncomplicated malaria and from disease controls. In the comparisons involving cerebral malaria (CM), we note that proteomics data seems to be weak in three of the four cases, and its seems that K+SCCA is hampered by this: Although it significantly improves over PCA-proteomics, it loses out to Raw-Clinical.

Another observation of the experiments is that K+SCCA benefits the prediction of the difficult class pairs more than the easier ones: in all of the comparisons where Raw-Clinical accuracy is below 85%, K+SCCA improves on the Raw-Clinical model.

Finally, we note that the canonical correlation analysis can also be used in the opposite way, namely to use the clinical data to extract more predictive biomarkers from proteomics data, and thus enhance the understanding of the systems biology underlying the complex phenotypes. Although this particular application was not the main focus in the present work, in our experiments the K+SCCA method often improved over the accuracy obtained with proteomics data alone.
